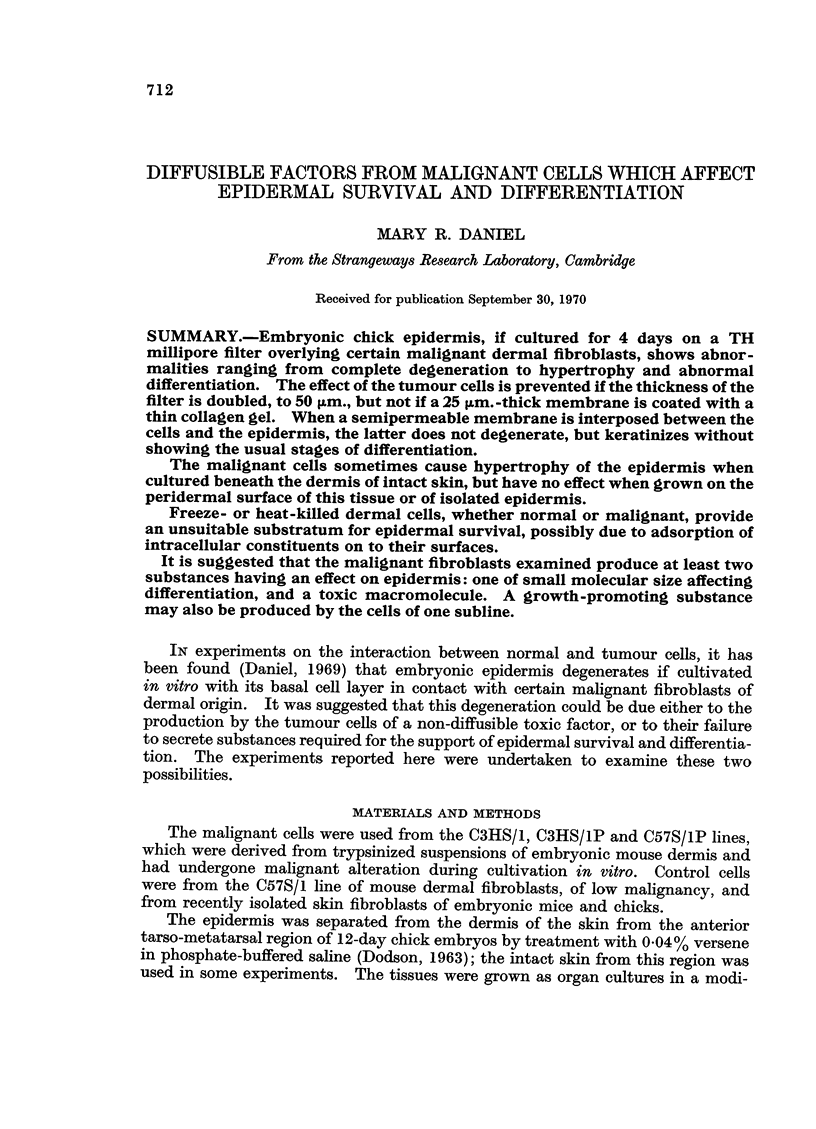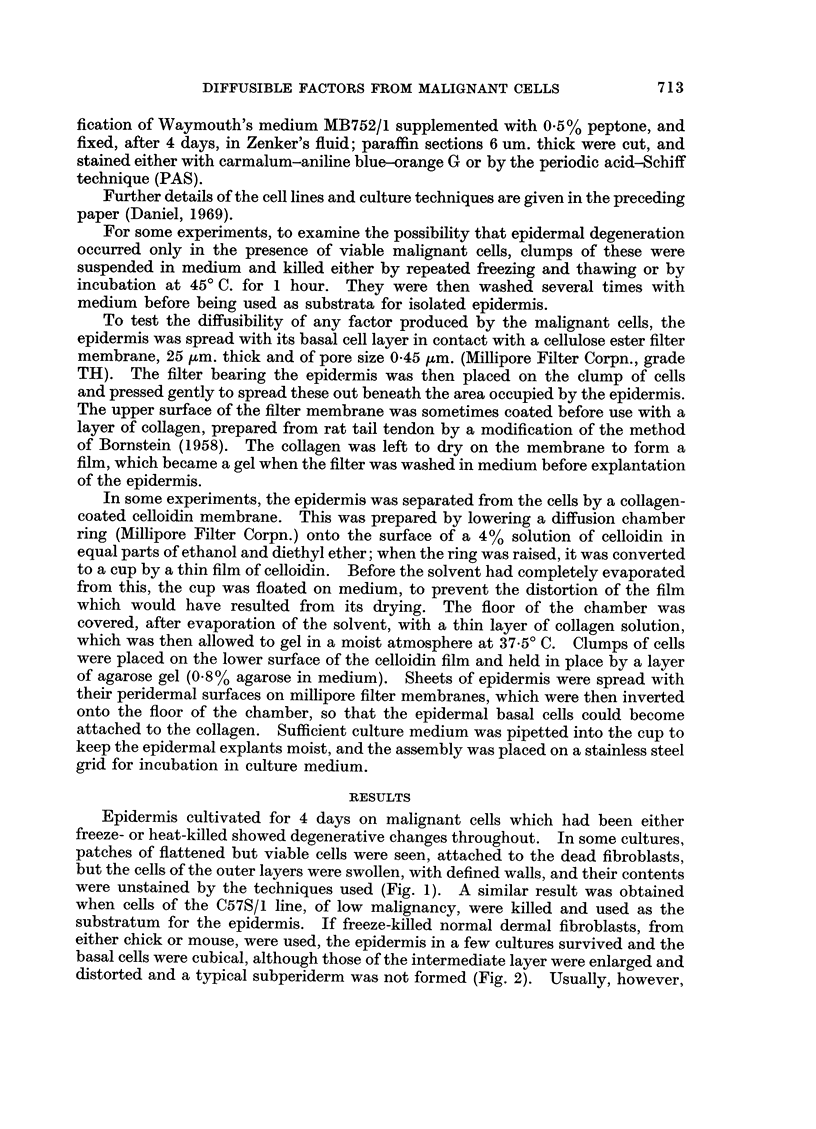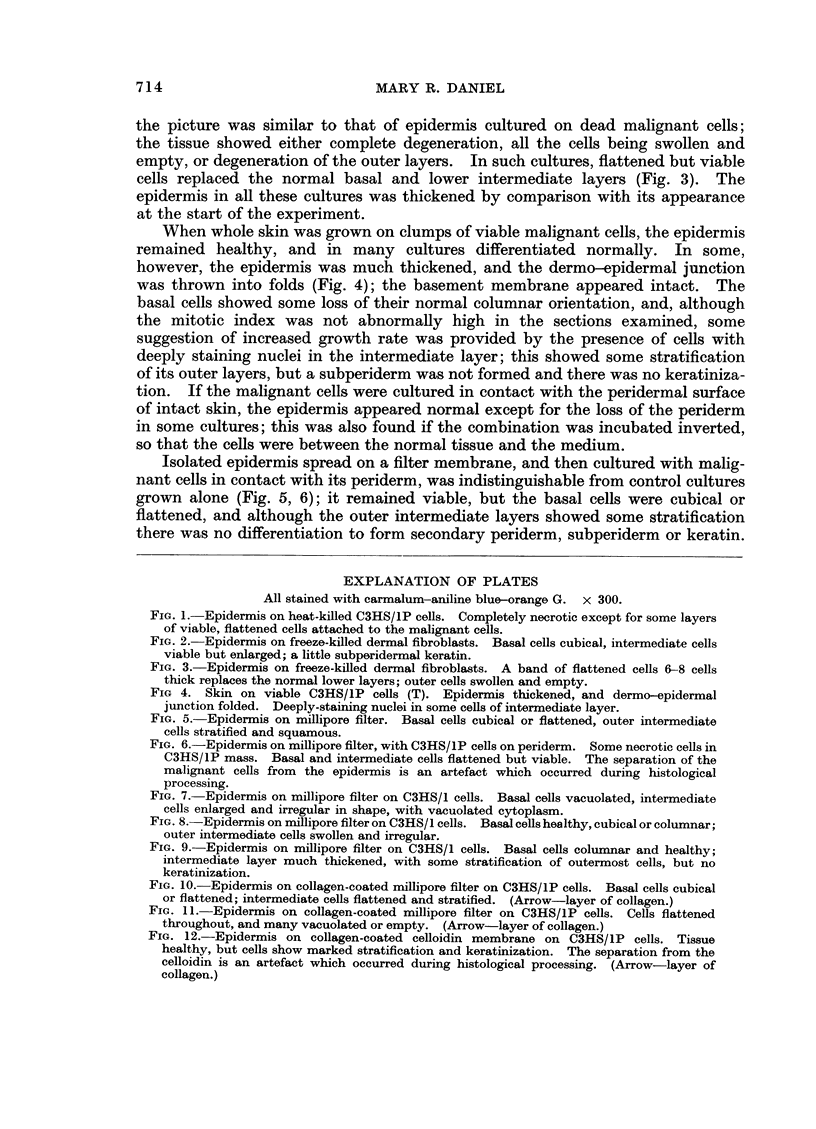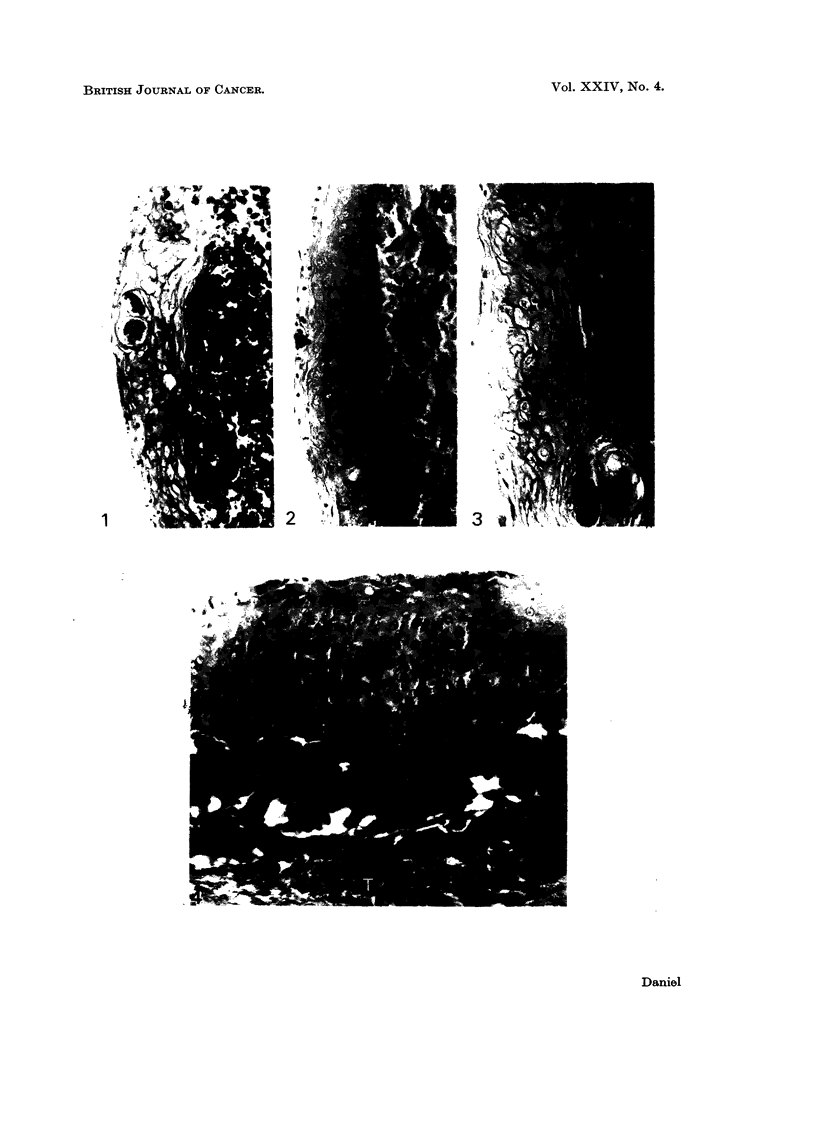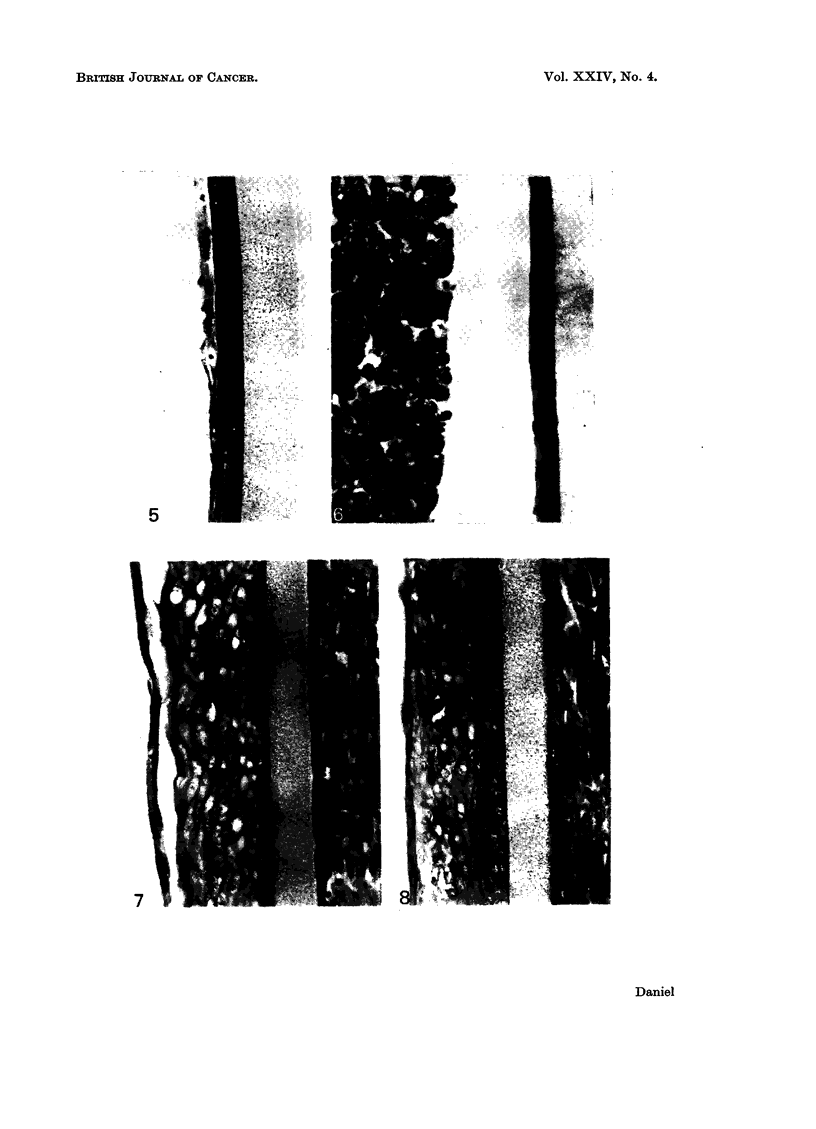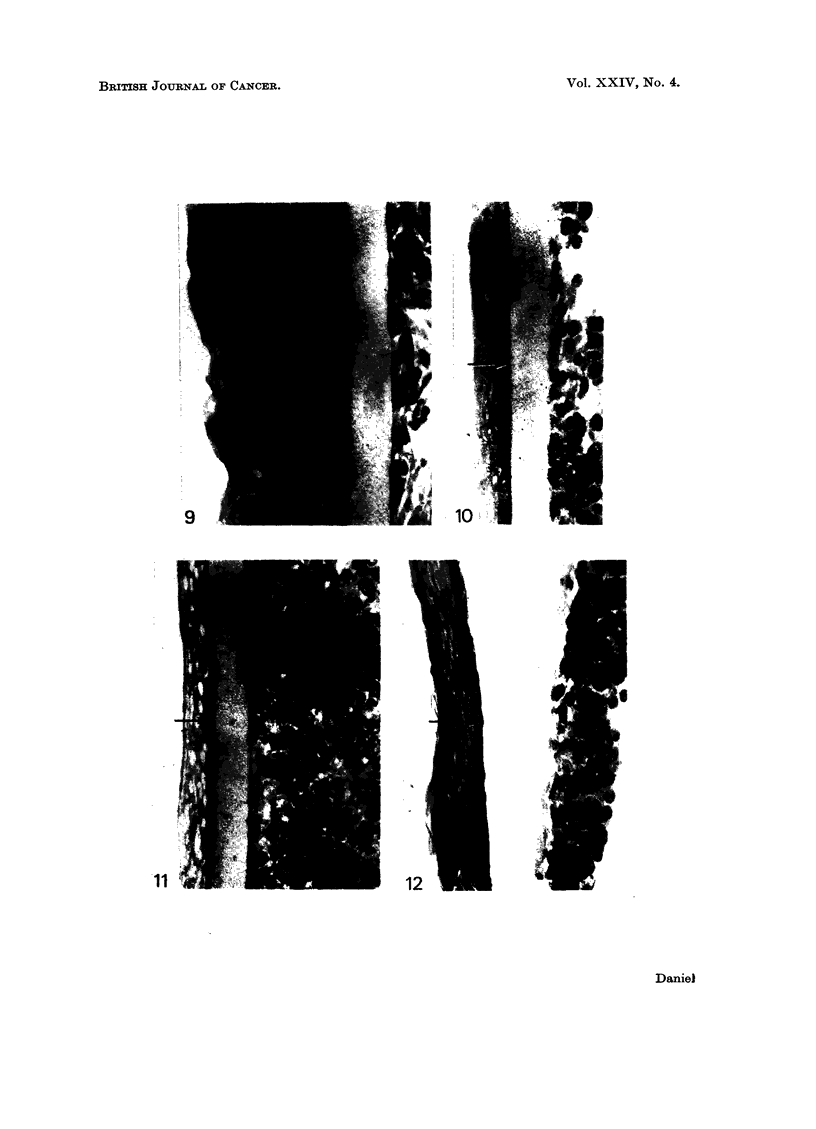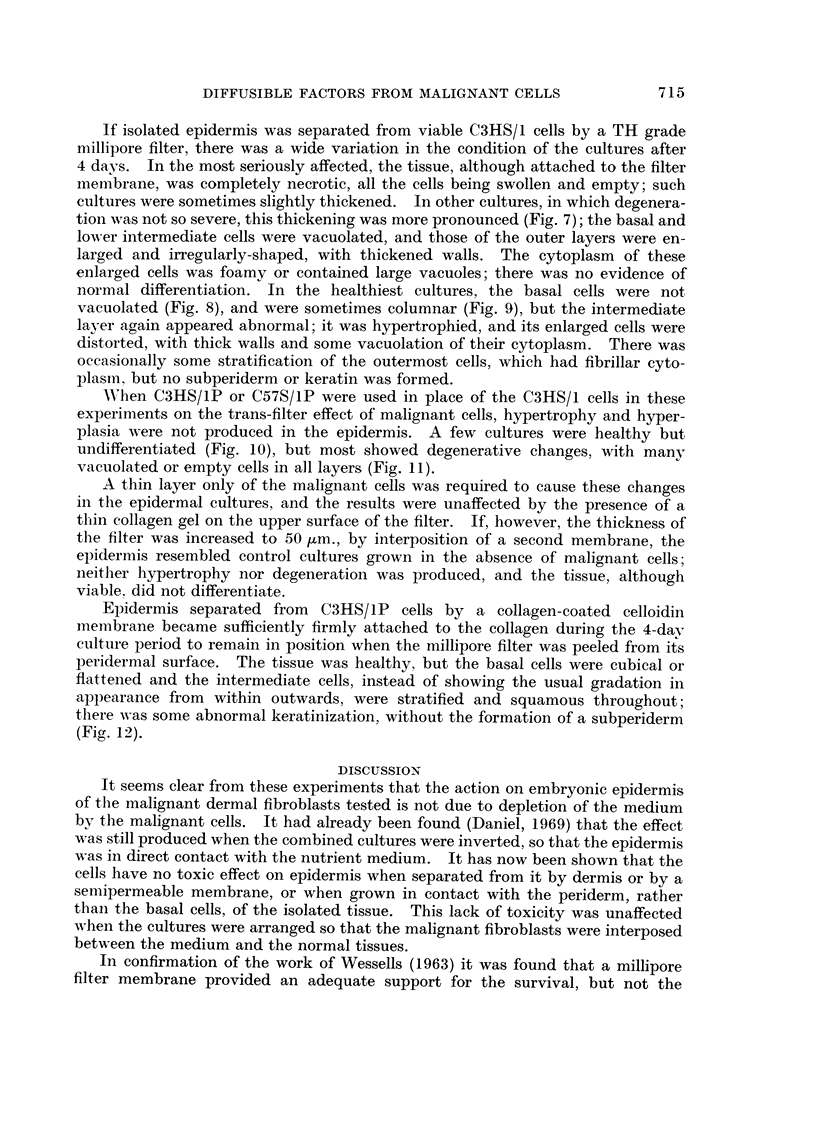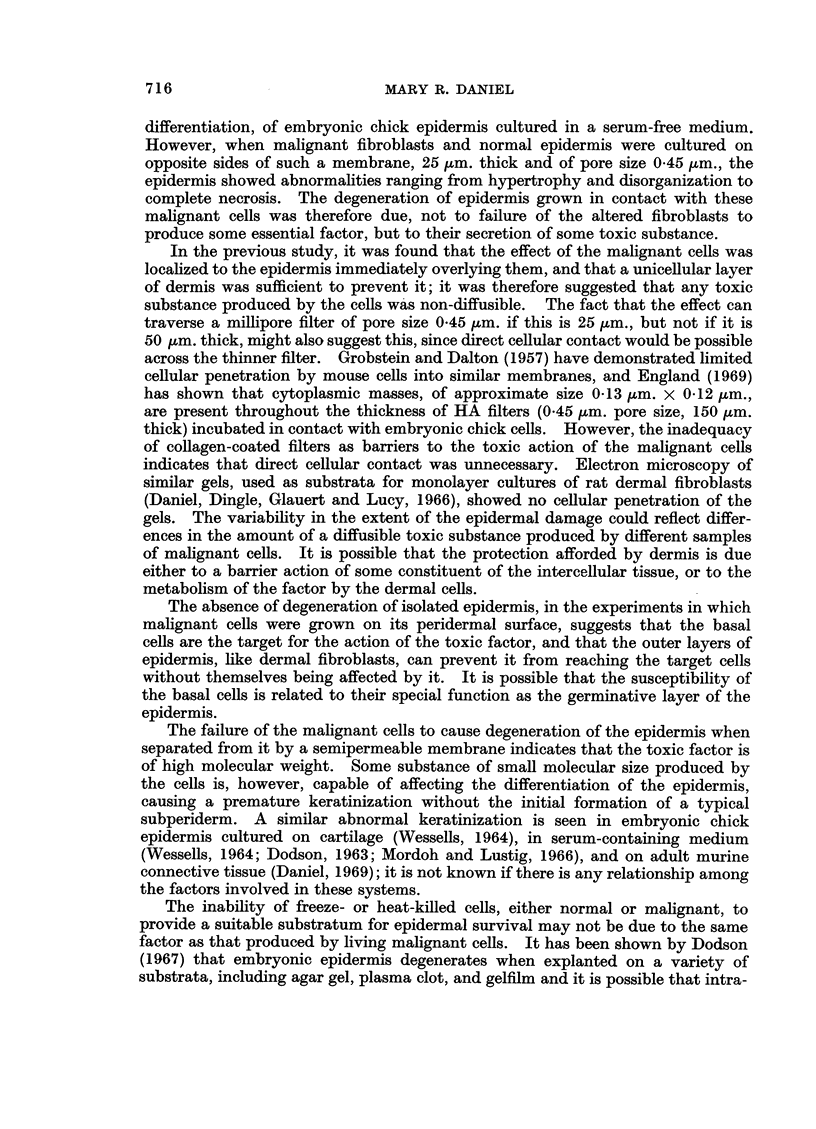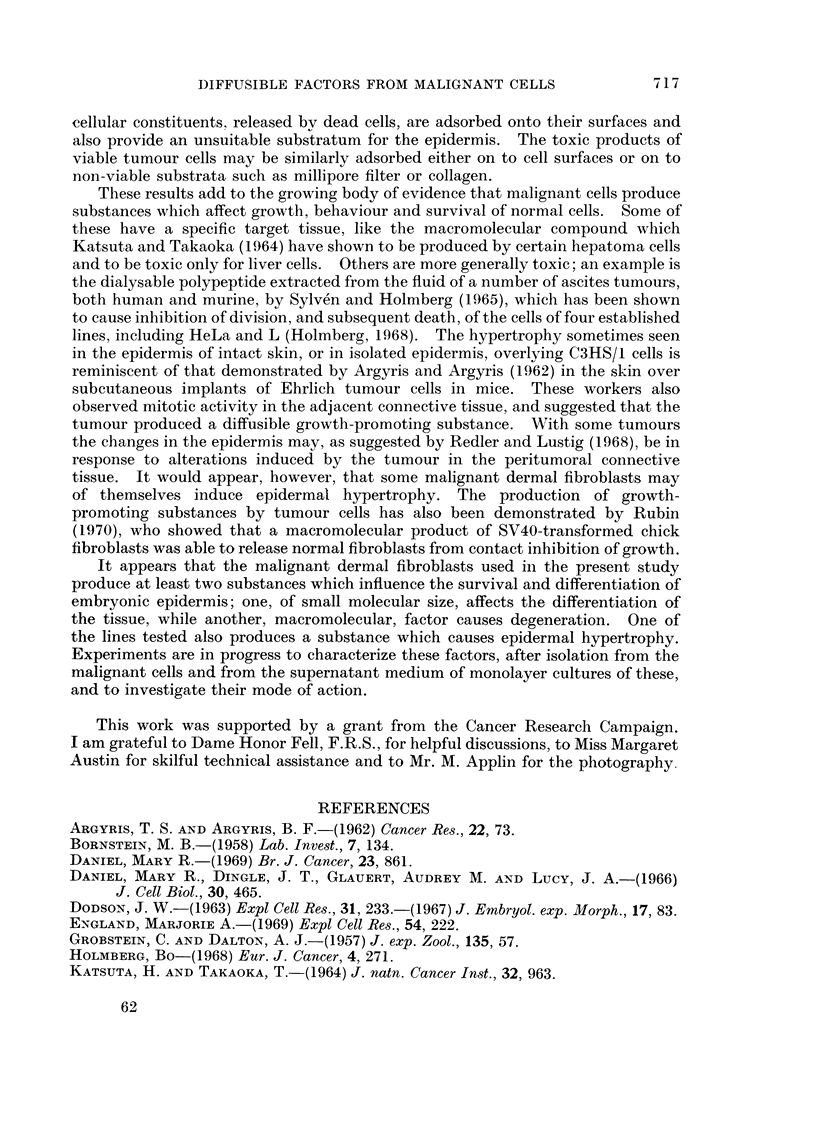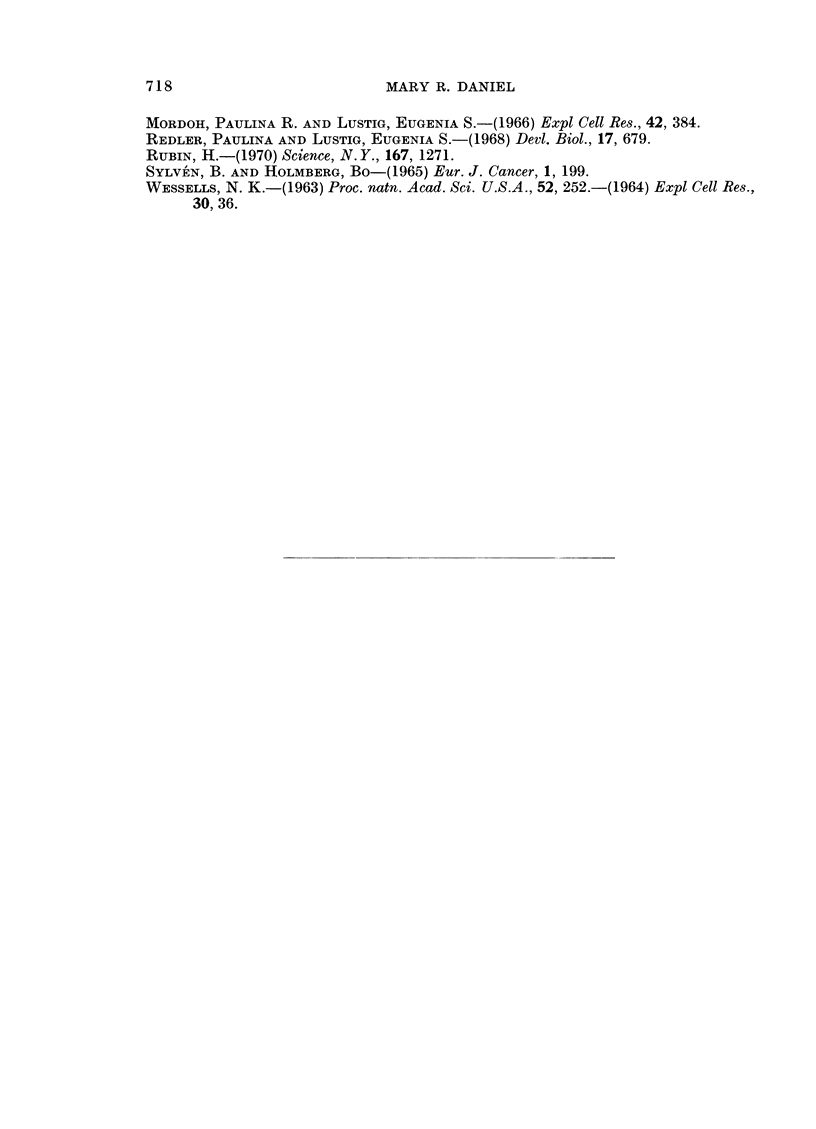# Diffusible Factors from Malignant Cells which Affect Epidermal Survival and Differentiation

**DOI:** 10.1038/bjc.1970.85

**Published:** 1970-12

**Authors:** Mary R. Daniel

## Abstract

**Images:**


### DIFFUSIBLE FACTORS FROM MALIGNANT CELLS WHICH AFFECT EPIDERMAL SURVIVAL AND DIFFERENTIATION

MARY R. DANIEL

*From the Strangeways Research Laboratory, Cambridge*

Received for publication September 30, 1970

**SUMMARY.—Embryonic chick epidermis, if cultured for 4 days on a TH millipore filter overlying certain malignant dermal fibroblasts, shows abnormalities ranging from complete degeneration to hypertrophy and abnormal differentiation. The effect of the tumour cells is prevented if the thickness of the filter is doubled, to 50 μm., but not if a 25 μm.-thick membrane is coated with a thin collagen gel. When a semipermeable membrane is interposed between the cells and the epidermis, the latter does not degenerate, but keratinizes without showing the usual stages of differentiation.**

**The malignant cells sometimes cause hypertrophy of the epidermis when cultured beneath the dermis of intact skin, but have no effect when grown on the peridermal surface of this tissue or of isolated epidermis.**

**Freeze- or heat-killed dermal cells, whether normal or malignant, provide an unsuitable substratum for epidermal survival, possibly due to adsorption of intracellular constituents on to their surfaces.**

**It is suggested that the malignant fibroblasts examined produce at least two substances having an effect on epidermis: one of small molecular size affecting differentiation, and a toxic macromolecule. A growth-promoting substance may also be produced by the cells of one subline.**

In experiments on the interaction between normal and tumour cells, it has been found (Daniel, 1969) that embryonic epidermis degenerates if cultivated *in vitro* with its basal cell layer in contact with certain malignant fibroblasts of dermal origin. It was suggested that this degeneration could be due either to the production by the tumour cells of a non-diffusible toxic factor, or to their failure to secrete substances required for the support of epidermal survival and differentiation. The experiments reported here were undertaken to examine these two possibilities.

### MATERIALS AND METHODS

The malignant cells were used from the C3HS/1, C3HS/1P and C57S/1P lines, which were derived from trypsinized suspensions of embryonic mouse dermis and had undergone malignant alteration during cultivation *in vitro*. Control cells were from the C57S/1 line of mouse dermal fibroblasts, of low malignancy, and from recently isolated skin fibroblasts of embryonic mice and chicks.

The epidermis was separated from the dermis of the skin from the anterior tarso-metatarsal region of 12-day chick embryos by treatment with 0·04% versene in phosphate-buffered saline (Dodson, 1963); the intact skin from this region was used in some experiments. The tissues were grown as organ cultures in a modification of Waymouth's medium MB752/1 supplemented with 0·5% peptone, and fixed, after 4 days, in Zenker's fluid; paraffin sections 6 um. thick were cut, and stained either with carmalum–aniline blue–orange G or by the periodic acid–Schiff technique (PAS).

Further details of the cell lines and culture techniques are given in the preceding paper (Daniel, 1969).

For some experiments, to examine the possibility that epidermal degeneration occurred only in the presence of viable malignant cells, clumps of these were suspended in medium and killed either by repeated freezing and thawing or by incubation at 45° C. for 1 hour. They were then washed several times with medium before being used as substrata for isolated epidermis.

To test the diffusibility of any factor produced by the malignant cells, the epidermis was spread with its basal cell layer in contact with a cellulose ester filter membrane, 25 $\mu$m. thick and of pore size 0·45 $\mu$m. (Millipore Filter Corpn., grade TH). The filter bearing the epidermis was then placed on the clump of cells and pressed gently to spread these out beneath the area occupied by the epidermis. The upper surface of the filter membrane was sometimes coated before use with a layer of collagen, prepared from rat tail tendon by a modification of the method of Bornstein (1958). The collagen was left to dry on the membrane to form a film, which became a gel when the filter was washed in medium before explantation of the epidermis.

In some experiments, the epidermis was separated from the cells by a collagen-coated celloidin membrane. This was prepared by lowering a diffusion chamber ring (Millipore Filter Corpn.) onto the surface of a 4% solution of celloidin in equal parts of ethanol and diethyl ether; when the ring was raised, it was converted to a cup by a thin film of celloidin. Before the solvent had completely evaporated from this, the cup was floated on medium, to prevent the distortion of the film which would have resulted from its drying. The floor of the chamber was covered, after evaporation of the solvent, with a thin layer of collagen solution, which was then allowed to gel in a moist atmosphere at 37·5° C. Clumps of cells were placed on the lower surface of the celloidin film and held in place by a layer of agarose gel (0·8% agarose in medium). Sheets of epidermis were spread with their peridermal surfaces on millipore filter membranes, which were then inverted onto the floor of the chamber, so that the epidermal basal cells could become attached to the collagen. Sufficient culture medium was pipetted into the cup to keep the epidermal explants moist, and the assembly was placed on a stainless steel grid for incubation in culture medium.

### RESULTS

Epidermis cultivated for 4 days on malignant cells which had been either freeze- or heat-killed showed degenerative changes throughout. In some cultures, patches of flattened but viable cells were seen, attached to the dead fibroblasts, but the cells of the outer layers were swollen, with defined walls, and their contents were unstained by the techniques used (Fig. 1). A similar result was obtained when cells of the C57S/1 line, of low malignancy, were killed and used as the substratum for the epidermis. If freeze-killed normal dermal fibroblasts, from either chick or mouse, were used, the epidermis in a few cultures survived and the basal cells were cubical, although those of the intermediate layer were enlarged and distorted and a typical subperiderm was not formed (Fig. 2). Usually, however, the picture was similar to that of epidermis cultured on dead malignant cells; the tissue showed either complete degeneration, all the cells being swollen and empty, or degeneration of the outer layers. In such cultures, flattened but viable cells replaced the normal basal and lower intermediate layers (Fig. 3). The epidermis in all these cultures was thickened by comparison with its appearance at the start of the experiment.

When whole skin was grown on clumps of viable malignant cells, the epidermis remained healthy, and in many cultures differentiated normally. In some, however, the epidermis was much thickened, and the dermo-epidermal junction was thrown into folds (Fig. 4); the basement membrane appeared intact. The basal cells showed some loss of their normal columnar orientation, and, although the mitotic index was not abnormally high in the sections examined, some suggestion of increased growth rate was provided by the presence of cells with deeply staining nuclei in the intermediate layer; this showed some stratification of its outer layers, but a subperiderm was not formed and there was no keratinization. If the malignant cells were cultured in contact with the peridermal surface of intact skin, the epidermis appeared normal except for the loss of the periderm in some cultures; this was also found if the combination was incubated inverted, so that the cells were between the normal tissue and the medium.

Isolated epidermis spread on a filter membrane, and then cultured with malignant cells in contact with its periderm, was indistinguishable from control cultures grown alone (Fig. 5, 6); it remained viable, but the basal cells were cubical or flattened, and although the outer intermediate layers showed some stratification there was no differentiation to form secondary periderm, subperiderm or keratin.

---

EXPLANATION OF PLATES

All stained with carmalum-aniline blue-orange G. × 300.

FIG. 1.—Epidermis on heat-killed C3HS/1P cells. Completely necrotic except for some layers of viable, flattened cells attached to the malignant cells.

FIG. 2.—Epidermis on freeze-killed dermal fibroblasts. Basal cells cubical, intermediate cells viable but enlarged; a little subperidermal keratin.

FIG. 3.—Epidermis on freeze-killed dermal fibroblasts. A band of flattened cells 6–8 cells thick replaces the normal lower layers; outer cells swollen and empty.

FIG 4. Skin on viable C3HS/1P cells (T). Epidermis thickened, and dermo-epidermal junction folded. Deeply-staining nuclei in some cells of intermediate layer.

FIG. 5.—Epidermis on millipore filter. Basal cells cubical or flattened, outer intermediate cells stratified and squamous.

FIG. 6.—Epidermis on millipore filter, with C3HS/1P cells on periderm. Some necrotic cells in C3HS/1P mass. Basal and intermediate cells flattened but viable. The separation of the malignant cells from the epidermis is an artefact which occurred during histological processing.

FIG. 7.—Epidermis on millipore filter on C3HS/1 cells. Basal cells vacuolated, intermediate cells enlarged and irregular in shape, with vacuolated cytoplasm.

FIG. 8.—Epidermis on millipore filter on C3HS/1 cells. Basal cells healthy, cubical or columnar; outer intermediate cells swollen and irregular.

FIG. 9.—Epidermis on millipore filter on C3HS/1 cells. Basal cells columnar and healthy; intermediate layer much thickened, with some stratification of outermost cells, but no keratinization.

FIG. 10.—Epidermis on collagen-coated millipore filter on C3HS/1P cells. Basal cells cubical or flattened; intermediate cells flattened and stratified. (Arrow—layer of collagen.)

FIG. 11.—Epidermis on collagen-coated millipore filter on C3HS/1P cells. Cells flattened throughout, and many vacuolated or empty. (Arrow—layer of collagen.)

FIG. 12.—Epidermis on collagen-coated celloidin membrane on C3HS/1P cells. Tissue healthy, but cells show marked stratification and keratinization. The separation from the celloidin is an artefact which occurred during histological processing. (Arrow—layer of collagen.)

Daniel

Daniel

Daniel

If isolated epidermis was separated from viable C3HS/1 cells by a TH grade millipore filter, there was a wide variation in the condition of the cultures after 4 days. In the most seriously affected, the tissue, although attached to the filter membrane, was completely necrotic, all the cells being swollen and empty; such cultures were sometimes slightly thickened. In other cultures, in which degeneration was not so severe, this thickening was more pronounced (Fig. 7); the basal and lower intermediate cells were vacuolated, and those of the outer layers were enlarged and irregularly-shaped, with thickened walls. The cytoplasm of these enlarged cells was foamy or contained large vacuoles; there was no evidence of normal differentiation. In the healthiest cultures, the basal cells were not vacuolated (Fig. 8), and were sometimes columnar (Fig. 9), but the intermediate layer again appeared abnormal; it was hypertrophied, and its enlarged cells were distorted, with thick walls and some vacuolation of their cytoplasm. There was occasionally some stratification of the outermost cells, which had fibrillar cytoplasm, but no subperiderm or keratin was formed.

When C3HS/1P or C57S/1P were used in place of the C3HS/1 cells in these experiments on the trans-filter effect of malignant cells, hypertrophy and hyperplasia were not produced in the epidermis. A few cultures were healthy but undifferentiated (Fig. 10), but most showed degenerative changes, with many vacuolated or empty cells in all layers (Fig. 11).

A thin layer only of the malignant cells was required to cause these changes in the epidermal cultures, and the results were unaffected by the presence of a thin collagen gel on the upper surface of the filter. If, however, the thickness of the filter was increased to 50 $\mu$m., by interposition of a second membrane, the epidermis resembled control cultures grown in the absence of malignant cells; neither hypertrophy nor degeneration was produced, and the tissue, although viable, did not differentiate.

Epidermis separated from C3HS/1P cells by a collagen-coated celloidin membrane became sufficiently firmly attached to the collagen during the 4-day culture period to remain in position when the millipore filter was peeled from its peridermal surface. The tissue was healthy, but the basal cells were cubical or flattened and the intermediate cells, instead of showing the usual gradation in appearance from within outwards, were stratified and squamous throughout; there was some abnormal keratinization, without the formation of a subperiderm (Fig. 12).

### DISCUSSION

It seems clear from these experiments that the action on embryonic epidermis of the malignant dermal fibroblasts tested is not due to depletion of the medium by the malignant cells. It had already been found (Daniel, 1969) that the effect was still produced when the combined cultures were inverted, so that the epidermis was in direct contact with the nutrient medium. It has now been shown that the cells have no toxic effect on epidermis when separated from it by dermis or by a semipermeable membrane, or when grown in contact with the periderm, rather than the basal cells, of the isolated tissue. This lack of toxicity was unaffected when the cultures were arranged so that the malignant fibroblasts were interposed between the medium and the normal tissues.

In confirmation of the work of Wessells (1963) it was found that a millipore filter membrane provided an adequate support for the survival, but not the differentiation, of embryonic chick epidermis cultured in a serum-free medium. However, when malignant fibroblasts and normal epidermis were cultured on opposite sides of such a membrane, 25 $\mu$m. thick and of pore size 0·45 $\mu$m., the epidermis showed abnormalities ranging from hypertrophy and disorganization to complete necrosis. The degeneration of epidermis grown in contact with these malignant cells was therefore due, not to failure of the altered fibroblasts to produce some essential factor, but to their secretion of some toxic substance.

In the previous study, it was found that the effect of the malignant cells was localized to the epidermis immediately overlying them, and that a unicellular layer of dermis was sufficient to prevent it; it was therefore suggested that any toxic substance produced by the cells was non-diffusible. The fact that the effect can traverse a millipore filter of pore size 0·45 $\mu$m. if this is 25 $\mu$m., but not if it is 50 $\mu$m. thick, might also suggest this, since direct cellular contact would be possible across the thinner filter. Grobstein and Dalton (1957) have demonstrated limited cellular penetration by mouse cells into similar membranes, and England (1969) has shown that cytoplasmic masses, of approximate size 0·13 $\mu$m. $\times$ 0·12 $\mu$m., are present throughout the thickness of HA filters (0·45 $\mu$m. pore size, 150 $\mu$m. thick) incubated in contact with embryonic chick cells. However, the inadequacy of collagen-coated filters as barriers to the toxic action of the malignant cells indicates that direct cellular contact was unnecessary. Electron microscopy of similar gels, used as substrata for monolayer cultures of rat dermal fibroblasts (Daniel, Dingle, Glauert and Lucy, 1966), showed no cellular penetration of the gels. The variability in the extent of the epidermal damage could reflect differences in the amount of a diffusible toxic substance produced by different samples of malignant cells. It is possible that the protection afforded by dermis is due either to a barrier action of some constituent of the intercellular tissue, or to the metabolism of the factor by the dermal cells.

The absence of degeneration of isolated epidermis, in the experiments in which malignant cells were grown on its peridermal surface, suggests that the basal cells are the target for the action of the toxic factor, and that the outer layers of epidermis, like dermal fibroblasts, can prevent it from reaching the target cells without themselves being affected by it. It is possible that the susceptibility of the basal cells is related to their special function as the germinative layer of the epidermis.

The failure of the malignant cells to cause degeneration of the epidermis when separated from it by a semipermeable membrane indicates that the toxic factor is of high molecular weight. Some substance of small molecular size produced by the cells is, however, capable of affecting the differentiation of the epidermis, causing a premature keratinization without the initial formation of a typical subperiderm. A similar abnormal keratinization is seen in embryonic chick epidermis cultured on cartilage (Wessells, 1964), in serum-containing medium (Wessells, 1964; Dodson, 1963; Mordoh and Lustig, 1966), and on adult murine connective tissue (Daniel, 1969); it is not known if there is any relationship among the factors involved in these systems.

The inability of freeze- or heat-killed cells, either normal or malignant, to provide a suitable substratum for epidermal survival may not be due to the same factor as that produced by living malignant cells. It has been shown by Dodson (1967) that embryonic epidermis degenerates when explanted on a variety of substrata, including agar gel, plasma clot, and gelfilm and it is possible that intracellular constituents, released by dead cells, are adsorbed onto their surfaces and also provide an unsuitable substratum for the epidermis. The toxic products of viable tumour cells may be similarly adsorbed either on to cell surfaces or on to non-viable substrata such as millipore filter or collagen.

These results add to the growing body of evidence that malignant cells produce substances which affect growth, behaviour and survival of normal cells. Some of these have a specific target tissue, like the macromolecular compound which Katsuta and Takaoka (1964) have shown to be produced by certain hepatoma cells and to be toxic only for liver cells. Others are more generally toxic; an example is the dialysable polypeptide extracted from the fluid of a number of ascites tumours, both human and murine, by Sylvén and Holmberg (1965), which has been shown to cause inhibition of division, and subsequent death, of the cells of four established lines, including HeLa and L (Holmberg, 1968). The hypertrophy sometimes seen in the epidermis of intact skin, or in isolated epidermis, overlying C3HS/1 cells is reminiscent of that demonstrated by Argyris and Argyris (1962) in the skin over subcutaneous implants of Ehrlich tumour cells in mice. These workers also observed mitotic activity in the adjacent connective tissue, and suggested that the tumour produced a diffusible growth-promoting substance. With some tumours the changes in the epidermis may, as suggested by Redler and Lustig (1968), be in response to alterations induced by the tumour in the peritumoral connective tissue. It would appear, however, that some malignant dermal fibroblasts may of themselves induce epidermal hypertrophy. The production of growth-promoting substances by tumour cells has also been demonstrated by Rubin (1970), who showed that a macromolecular product of SV40-transformed chick fibroblasts was able to release normal fibroblasts from contact inhibition of growth.

It appears that the malignant dermal fibroblasts used in the present study produce at least two substances which influence the survival and differentiation of embryonic epidermis; one, of small molecular size, affects the differentiation of the tissue, while another, macromolecular, factor causes degeneration. One of the lines tested also produces a substance which causes epidermal hypertrophy. Experiments are in progress to characterize these factors, after isolation from the malignant cells and from the supernatant medium of monolayer cultures of these, and to investigate their mode of action.

This work was supported by a grant from the Cancer Research Campaign. I am grateful to Dame Honor Fell, F.R.S., for helpful discussions, to Miss Margaret Austin for skilful technical assistance and to Mr. M. Applin for the photography.

### REFERENCES

Argyris, T. S. and Argyris, B. F.—(1962) *Cancer Res.*, **22**, 73.
Bornstein, M. B.—(1958) *Lab. Invest.*, **7**, 134.
Daniel, Mary R.—(1969) *Br. J. Cancer*, **23**, 861.
Daniel, Mary R., Dingle, J. T., Glauert, Audrey M. and Lucy, J. A.—(1966) *J. Cell Biol.*, **30**, 465.
Dodson, J. W.—(1963) *Expl Cell Res.*, **31**, 233.—(1967) *J. Embryol. exp. Morph.*, **17**, 83.
England, Marjorie A.—(1969) *Expl Cell Res.*, **54**, 222.
Grobstein, C. and Dalton, A. J.—(1957) *J. exp. Zool.*, **135**, 57.
Holmberg, Bo—(1968) *Eur. J. Cancer*, **4**, 271.
Katsuta, H. and Takaoka, T.—(1964) *J. natn. Cancer Inst.*, **32**, 963.

Mordoh, Paulina R. and Lustig, Eugenia S.—(1966) *Expl Cell Res.*, **42**, 384.
Redler, Paulina and Lustig, Eugenia S.—(1968) *Devl. Biol.*, **17**, 679.
Rubin, H.—(1970) *Science, N.Y.*, **167**, 1271.
Sylvén, B. and Holmberg, Bo—(1965) *Eur. J. Cancer*, **1**, 199.
Wessells, N. K.—(1963) *Proc. natn. Acad. Sci. U.S.A.*, **52**, 252.—(1964) *Expl Cell Res.*, **30**, 36.